# Analysis of the main components of LSPN based on broadly targeted metabolomics technology

**DOI:** 10.3389/fpls.2025.1618168

**Published:** 2025-10-22

**Authors:** Feng Qin, Yuyong Chen, Dada Wang, Jingui Li, Shanyuan Zhu

**Affiliations:** ^1^ Jiangsu Key Laboratory for High-Tech Research and Development of Veterinary Biopharmaceuticals, Engineering Technology Research Center for Modern Animal Science and Novel Veterinary Pharmaceutic Development, Jiangsu Agri-Animal Husbandry Vocational College, Taizhou, Jiangsu, China; ^2^ College of Veterinary Medicine, Yangzhou University, Yangzhou, Jiangsu, China; ^3^ College of Food Science and Technology, Jiangsu Agri-animal Husbandry Vocational College, Taizhou, Jiangsu, China; ^4^ College of Animal Pharmaceutical, Jiangsu Agri-animal Husbandry Vocational College, Taizhou, Jiangsu, China

**Keywords:** targeted metabolome, *Panax notoginseng* stems and leaves, triterpenoid saponins, biological activity, main components

## Abstract

**Introduction:**

This study aimed to (1) establish a broadly targeted metabolomics workflow to evaluate batch-to-batch variability in *Panax notoginseng*-derived *Panax notoginseng* stem/leaf-derived oral liquid (LSPN) preparations, (2) identify critical process-induced metabolite differences (e.g., saponin degradation products), and (3) correlate these differences with pharmacopoeial quality markers (e.g., Rg1/Rb1 ratios).

**Methods:**

Our hypothesis was that thermal extraction parameters would significantly alter saponin profiles, detectable via orthogonal metabolomic approaches (HPLC-MS/MS, PCA).

**Results:**

The results showed a strong positive correlation (pearson correlation coefficient >0.92) between triterpenoid saponins in LSPN-S and LSPN-C. Thirty-eight triterpenoid saponins were detected, of which 11 were up-regulated, 5 were down-regulated, and the other 22 had less than a two-fold difference in content. Ginsenoside Rb3 showed a modest but significant downregulation in LSPN-S (fold change = 0.45, VIP = 1.11) compared with LSPN-C.

**Discussion:**

Metabolomic analysis confirmed the suitability of the selected *Panax notoginseng* stems/leaves as raw materials, with comprehensive characterization of LSPN’s primary components.

## 1 Introduction

*Panax notoginseng* (Burk.) F. H. Chen stems and leaves are the dried stems and leaves of *Panax notoginseng* (Burk.) F. H. Chen in the Araliaceae family. “Compendium of Materia Medica”records that its effects are similar to those of the root, and has anti-depressant, anti-anxiety, analgesic, anti-inflammatory, anti-aging, regulating blood lipids, and protecting cardiovascular and cerebrovascular effects (Li et al., 2022a), and the immune-enhancing effects of Panax quinquefolium root have been widely demonstrated. Panax pseudoginseng stem and leaves are the by-products of the processing of Panax pseudoginseng, with a total annual output of about 2,500 tons, at a relatively low cost, rich in saponin compounds, with a total saponin content of about 8.53%; however, the utilization rate of Panax pseudoginseng stem and leaves is only about 5%. Based on the principle of “turning waste into treasure”, Panax pseudoginseng stem and leaves were selected as the object of study in the present study, and the *Panax notoginseng* stem/leaf oral liquid (LSPN) was prepared by using a pre-existing chitosan removal process, which was based on the principle of “turning waste into treasure”. The LSPN was prepared by using the pre-chitosan removal process (Zhang et al., 2020). In order to further identify whether the selected *Panax notoginseng* stems and leaves are suitable for use as raw medicinal materials for LSPN and analyze the main components of LSPN, a comparative study on the ingredients of *Panax notoginseng* stems and leaves is needed.

Plant metabolomics can characterize and quantify small molecule compounds in plant samples. Mass spectrometry and nuclear magnetic resonance are the main tools. In particular, mass spectrometry technology has the advantages of wide linear range, low cost, and high sensitivity (Xiao et al., 2024). A self-built database, MWDB (metware database), was used to qualitatively analyze the substances based on secondary spectra, and quantitatively analyze the substances using multiple reaction monitoring (MRM) mode (Liu et al., 2022). In the MRM model, the parent ion (precursor ion) of the target component is screened out first, the precursor ion ionizes and breaks off, and the resulting fragment ion passes through the triple quadruple quadruple bar again, selecting a characteristic fragment ion quantitatively (Li et al., 2018). After analyzing the metabolite mass spectrometry data of different samples, the peak areas of the mass spectrum peaks of all substances are integrated, and the mass spectrum peaks of the same metabolite in different samples are integrated and corrected (Li and Li, 2020).

**Figure 1:**
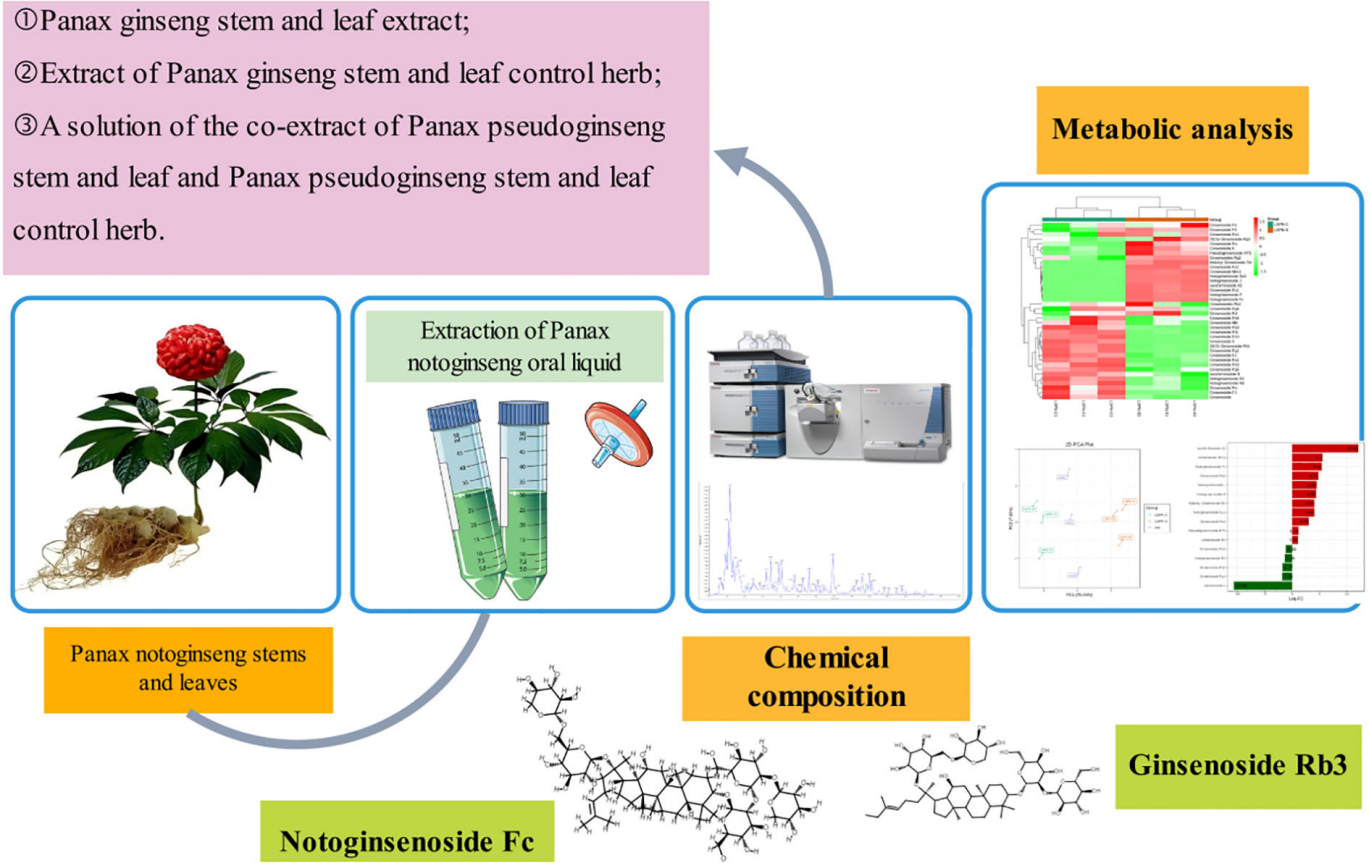
The overall flow chart of this study.

While metabolomics has been applied to Panax species (Bi-lian, 2013; Chen et al., 2013; Chen et al., 2019), no studies have systematically linked LSPN production variability to metabolomic signatures under standardized quality frameworks. Here, we test the hypothesis that batch-specific processing conditions (e.g., temperature) drive quantifiable differences in saponin profiles, which may compromise consistency in bioactivity. Using a tripartite analytical strategy—(i) untargeted metabolomics for discovery, (ii) targeted quantification of 20 key saponins, and (iii) bioactivity prediction via network pharmacology—we address three measurable outcomes: PCA clustering of LSPN-C vs. LSPN-S (acceptance threshold: >80% variance explained);≥2-fold changes in thermally labile saponins (e.g., Rg3, p < 0.01);Spearman’s rank (ρ > 0.7) between HPLC fingerprints and *in vitro* antiplatelet activity. In order to identify the main components of LSPN and explore the quality of oral liquid prepared from several selected batches of medicinal materials, this study intends to prepare different batches of LSPN based on the optimal process of preliminary screening, and compare it with the LSPN prepared from the reference medicinal materials according to the above process. Compare, analyze the differences in their ingredients, and comprehensively determine their quality (**Figure 1**).

## 2 Materials and methods

### 2.1 Standards and reagents

Methanol, acetonitrile and ethanol were chromatographically pure and purchased from Merck; the internal standard substance: 2-chlorophenylalanine (batch no.: LBCOR15) with 98% purity was purchased from BioBioPha/Sigma-Aldrich; Panax ginseng stem and leaf control herbs (batch nos.: FY20170112, FYD100801, FYD100702) were purchased from Nantong Feiyu Biotechnology Co. Ltd; Panax pseudoginseng stem/leaf medicinal materials, purchased from Yunnan Wenshan Panax pseudoginseng Research Institute.

For metabolomic profiling, all LSPN batches were analyzed using a Waters ACQUITY UPLC system coupled with a Xevo G2-XS QTOF mass spectrometer (positive/negative ESI mode; capillary voltage: 3.0 kV; desolvation gas flow: 800 L/h).

LSPN-C (control) and LSPN-S (experimental) samples were derived from distinct production batches with documented variations in critical processing parameters (e.g., extraction temperature: 70°Cvs. 90°C; duration: 2 h vs. 3 h). These batches were selected based on: (i) Significant differences in total saponin content (HPLC quantification, p < 0.05, ≥5% variation);(ii) Non-superimposable HPLC fingerprints (cosine similarity score <0.90), and (iii) Compliance with the Chinese Pharmacopoeia (2020 Edition) limits for notoginsenoside R1, ginsenoside Rg1, and Rb1 (Zheng et al., 2022). This grouping strategy aligns with industry standards for assessing batch-to-batch variability in herbal preparations (Szijj and Chudasama, 2021).

Regarding the sample size (n=3), it is important to clarify that each ‘n’ represents an independent biological replicate (a distinct production batch), not a technical replicate. In metabolomics studies, where the primary objective is to uncover systematic patterns and trends across different groups rather than comparing means between small groups, a sample size of 3–6 per group is common and accepted, especially when coupled with robust multivariate statistical models (like OPLS-DA) that are powerful in handling such data structures. While we acknowledge that a larger sample size would provide greater statistical power, the observed large fold changes (e.g., >20-fold for some saponins) and excellent model validation parameters (Q² = 0.995) provide high confidence that the differences we report are biologically significant and not due to random chance.

### 2.2 LC conditions​

The chromatographic separation was performed using an ACQUITY UPLC HSS T3 column (2.1 × 100 mm, 1.8 μm) maintained at 40 °C. The mobile phase consisted of 0.1% formic acid in water (A) and 0.1% formic acid in acetonitrile (B), delivered at a constant flow rate of 0.35 mL/min. The gradient program started at 5% B, increased linearly to 30% B over 2 min, then to 60% B by 10 min, followed by a rapid increase to 95% B at 12 min (held for 2 min), before re-equilibrating at 5% B for 2 min. The injection volume was 2 μL.

### 2.3 MS/MS conditions​

Mass spectrometric detection was performed using electrospray ionization (ESI) in positive/negative switching mode. The ion source parameters were optimized as follows: ion spray voltage +5500 V (positive) and -4500 V (negative), source temperature 550 °C, with gas flows of 50 psi (GS1), 60 psi (GS2), and 30 psi (CUR). For each saponin, specific multiple reaction monitoring (MRM) transitions were established after optimization of collision energy (CE) and declustering potential (DP). Key examples include Ginsenoside Rb1 (1131.6→365.2 m/z, CE 40 V, DP 120 V), Ginsenoside Rg1 (823.5→643.4 m/z, CE 35 V, DP 110 V), and Notoginsenoside R1 (954.5→789.4 m/z, CE 45 V, DP 130 V). The complete list of optimized MRM transitions for all 38 saponins is provided in **Supplementary Materials**.

### 2.4 Quality control and validation​

System suitability was monitored by injecting pooled QC samples every 10 runs, with acceptance criteria of <0.5% RSD for retention time and <15% RSD for peak area. Sensitivity was determined through serial dilution, establishing limits of detection (LOD, S/N ≥3) and quantification (LOQ, S/N ≥10) for each analyte.

### 2.5 Sample group preparation

Sample numbers and groupings for this experiment are shown in **Table 1**. Thaw the sample at -80 °Cand vortex for 10 s to mix. 100 uL of the mixed sample was taken and 100 uL of 70% methanol internal (Preliminary tests showing optimal saponin recovery; compatibility with our LC-MS/MS system; Consistency with Chinese Pharmacopoeia (2020) methods for similar compounds) standard extract was added. After vortexing for 3 min, the sample was centrifuged for 10 min (12000 r-min-1, 4 °C). The supernatant was filtered through a microporous membrane (0.22 μm) and stored in the injection bottle for LC-MS/MS detection.

**Table 1:** Sample grouping.

Tissue	Treatment	Specimen	Group
Panax pseudoginseng Stem and Leaf Control Herbs	Water Extract Oral Liquid	LSPN-C1	LSPN-C
Panax pseudoginseng Stem and Leaf Control Herbs	Water Extract Oral Liquid	LSPN-C2	LSPN-C
Panax pseudoginseng Stem and Leaf Control Herbs	Water Extract Oral Liquid	LSPN-C3	LSPN-C
Panax pseudoginseng stem/leaf medicinal materials	Water Extract Oral Liquid	LSPN-S1	LSPN-S
Panax pseudoginseng stem/leaf medicinal materials	Water Extract Oral Liquid	LSPN-S2	LSPN-S
Panax pseudoginseng stem/leaf medicinal materials	Water Extract Oral Liquid	LSPN-S3	LSPN-S
Panax pseudoginseng Stem and Leaf Mix	Extracts of an equal mass mixture of Panax pseudoginseng stem and leaf control herb and Panax pseudoginseng stem and leaf herb were used as quality control samples	mix01	mix
Panax pseudoginseng Stem and Leaf Mix	Extracts of an equal mass mixture of Panax pseudoginseng stem and leaf control herb and Panax pseudoginseng stem and leaf herb were used as quality control samples	mix02	mix
Panax pseudoginseng Stem and Leaf Mix	Extracts of an equal mass mixture of Panax pseudoginseng stem and leaf control herb and Panax pseudoginseng stem and leaf herb were used as quality control samples	mix03	mix

Pooled samples were injected every 10 runs to monitor instrument stability (RSD <20% for 90% of features). QC sample analyses demonstrated high spectral overlap (TIC plots), confirming method reproducibility.

Each independently prepared sample extract (a biological replicate, n=3 per group) was injected once for LC-MS/MS analysis. The exceptional reproducibility of our UPLC-MS/MS system, coupled with the use of a pooled quality control (QC) sample injected repeatedly throughout the analytical sequence, allowed us to confidently assess and ensure analytical precision without the need for technical replicates for each sample. This approach is well-established in targeted metabolomics studies where system stability is rigorously monitored.

### 2.6 Mass spectrometry conditions and identification of saponin components

Electrospray ionization (ESI) temperature is 550 °C, mass spectrometer voltage is 5500 V (positive ion mode) and -4500 V (negative ion mode), ion source gas I (ion source gas I, GSI) 50 psi, ion source gas II (ion source gas II, GSII) 60 psi, curtain gas (CUR) 30 psi, and collision-activated dissociation (CAD) parameters were set to high. Instrument tuning and mass calibration were performed using 10 and 100 μmol/L polypropylene glycol solutions in QQQ and LIT modes, respectively. In the triple quadrupole (QQQ), each ion pair is scanned and detected based on the optimized declustering potential (DP) and collision energy (CE) (Fraga et al., 2010). QQQ scans were performed in an MRM experiment with the collision gas (nitrogen) set to 5 psi. Based on the self-built database MWDB, the substance is characterized according to the secondary spectrum information and the MRM is used to integrate the peak area of the mass spectrum peaks of all substances, and the mass spectrum peaks of the same compound in different samples are Integration correction is performed, and the peak area represents the relative content of the substance in the sample.

Compound identification was performed using the MWDB database with the following thresholds, Precursor ion mass error: ≤ 5 ppm; Fragment ion mass error: ≤ 10 ppm; Spectral similarity score: ≥ 0.8 (cosine similarity); Minimum matched fragment ions: ≥ 5. Compounds failing any of these criteria were excluded from further analysis.

According to the confidence level guidelines proposed by the Metabolomics Standards Initiative (MSI), identifications are reported at two levels: Level 1 (confirmed by reference standard): 12 saponins (including Ginsenoside Rg1, Rb1, etc.) were unequivocally confirmed using commercially available chemical standards. Level 2 (putatively annotated based on spectral similarity): The remaining 26 compounds were identified by comparison to the high-resolution MS/MS spectral library (MWDB) with very strict matching criteria as stated above. While this provides a high degree of confidence, we acknowledge that definitive confirmation of these Level 2 identifications requires further purification and NMR analysis in future work.

The confidence levels for the identification of all detected saponins were classified according to the Metabolomics Standards Initiative (MSI) guidelines. Level 1 (Confidently identified): Twelve (Fraga et al., 2010) saponins (including Ginsenoside Rg1, Rb1, and Notoginsenoside R1) were unequivocally confirmed by comparison with authentic reference standards under identical analytical conditions (matching retention time and MS/MS spectra). Level 2 (Putatively annotated): The remaining twenty-six (Zhao et al., 2018) compounds were identified based on high-resolution MS/MS spectral matching with the self-built database (MWDB) using strict criteria: precursor ion mass error ≤ 5 ppm, fragment ion mass error ≤ 10 ppm, spectral similarity score (cosine similarity) ≥ 0.8, and a minimum of 5 matched fragment ions. While this approach provides a high degree of confidence for annotation, we acknowledge that definitive confirmation of these Level 2 identifications, particularly for isomeric saponins (e.g., Rg2/Rg6), requires further purification and structural elucidation by NMR spectroscopy in future studies.

### 2.7 Data analysis

R software (version 3.5.0) was used for multivariate statistical analysis. The KEGG database was also used for differential compound annotation and presentation (Ogata et al., 1999). RSD < 15% for retention time and peak area (n = 5 replicates);RSD < 20% (n = 3 days);Pooled samples injected every 10 runs to monitor instrument stability (RSD < 20% for 90% of features).

### 2.8 Method validation and replicability

Five replicate injections of a pooled QC sample within a single analytical run showed RSD <15% for retention time and peak area for all major saponins; Inter-day precision: Three replicate injections of the same QC sample across three consecutive days demonstrated RSD <20%; Acceptance criteria: Features with RSD <20% for RT and <25% for peak area were retained for further analysis.

Limit of detection (LOD) and limit of quantification (LOQ) were determined via serial dilution of saponin standards (Rg1, Rb1, R1), with LOD defined as signal-to-noise ratio (S/N) ≥3 and LOQ as S/N ≥10. Values ranged from 0.01-0.05 μg/mL (LOD) and 0.03-0.15 μg/mL (LOQ) across analytes.

The precision and stability of the analytical platform were rigorously monitored using the pooled QC sample. The low intra-day (RSD < 15%, n = 5 replicate injections of the QC within one day) and inter-day (RSD < 20%, n = 3 replicate injections of the QC across three consecutive days) variation demonstrated excellent instrumental reproducibility. Consequently, the variation observed in the biological replicates (n=3) primarily reflects true biological and preparative differences between batches, rather than analytical noise.

## 3 Results

### 3.1 Qualitative and quantitative analysis of the saponin-like components of LSPN

**Figure 2:**
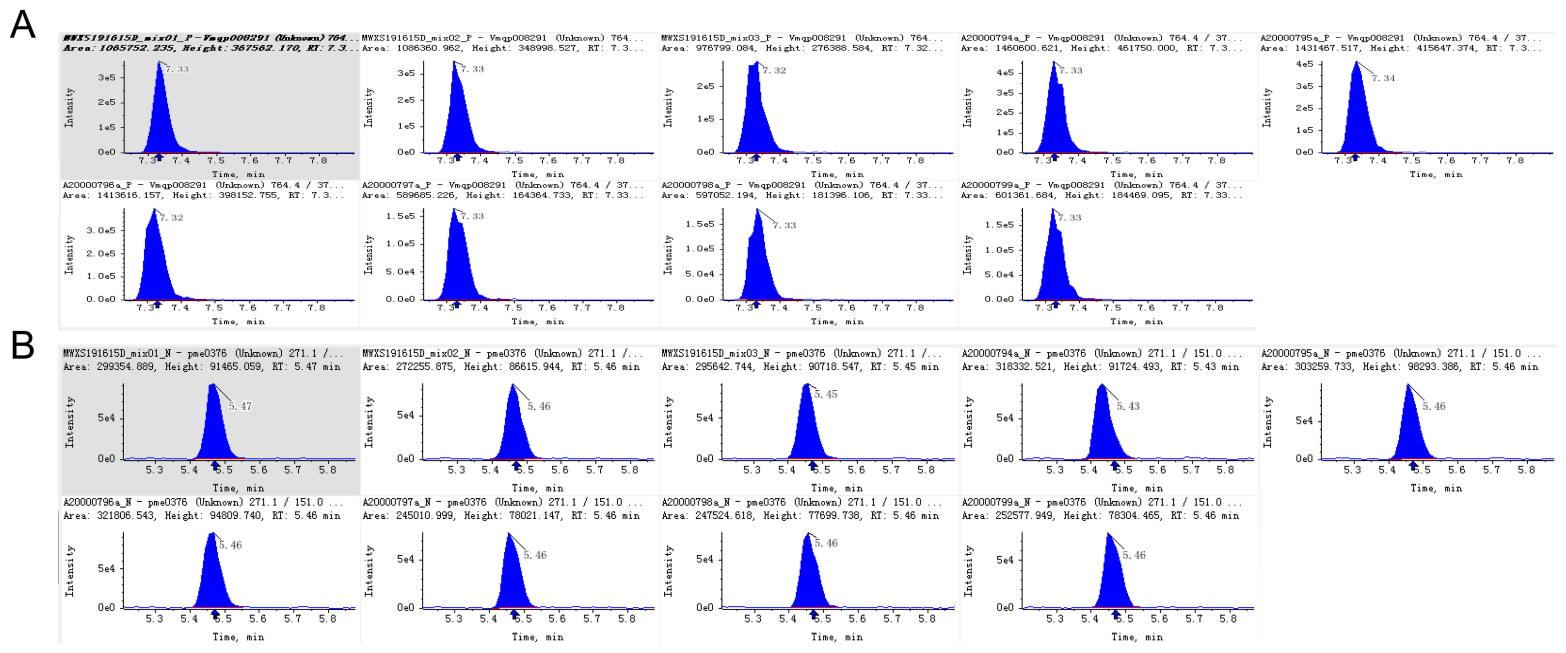
Integral correction chart of randomly selected metabolite quantitative analysis of different LSPN. **(A)** Positive Ion Mode; **(B)** Negative Ion Mode. The abscissa is the retention time (min) of the chemical compound, and the ordinate is the ion current intensity (cps) of the chemical compound. The peak area is proportional to the content of the compound.

After qualitative and quantitative analysis, the triterpene saponin species of Panax pseudoginseng stem and leaf control herb, Panax pseudoginseng stem and leaf herb, and the mixed quality control (QC) samples were identical; therefore, the mixed QC samples were used for demonstration of the total ion flow diagrams, TIC overlap diagrams, and types and structures of saponin compounds. The total ion flow plots are shown in **Supplementary Figure S1**. The multi-peak plots of MRM compound detection are shown in **Supplementary Figure S2**. The integral correction plots of quantitative analysis of randomly selected compounds are shown in **Figure 2**.

In addition, the main compounds in **Supplementary Figure S1** can be basically separated. In **Supplementary Figure S2**, each mass spectrometry peak of a different color represents a detected compound, and different compounds can be clearly distinguished. The integral correction plots of quantitative analysis of randomly selected compounds (**Figure 2**) showed small differences in peak areas (RSD <15%) across replicates, indicating good method resolution and stability.

Overlapping TIC plots of QC samples (**Table 1**; **Supplementary Figure S3**) confirmed signal stability and reproducibility. By comparing the compound spectral libraries, a total of 530 compounds were detected (data omitted), including 38 triterpenoid saponins, as shown in **Table 2**.

**Table 2:** Type and structural formula of saponin compounds detected in mixed samples.

ndex	Q1 (Da)	Q3 (Da)	Molecular weight (Da)	Formula	Ionization model	Compounds	Substance
HJAP107	621.44	423.36	620.429	C_36_H_60_O_8_	[M+H]+	Ginsenoside Rh4	Ginsenoside Rh4
Lmzn004497	1209.63	1149.6	1210.635	C_58_H_98_O_26_	[M-H]-	Notoginsenoside Fc	Ginsenoside Fc
Lmzp004417	639.45	441.37	638.439	C_36_H_62_O_9_	[M+H]+	Ginsenoside Rf1	Ginsenoside Rf1
Lmzp005197	767.49	407.37	766.487	C_42_H_70_O_12_	[M+H]+	Ginsenoside Rg5	Ginsenoside Rg5
Lmzp005427	947.56	325.11	946.55	C_48_H_82_O_18_	[M+H]+	Ginsenoside Rd	Ginsenoside Rd
MWKH438401	1077.58	945.5	1078.593	C_53_H_90_O_22_	[M-H]-	Ginsenoside Rb3	Ginsenoside Rb3
mws1553	799.48	637.4	800.492	C_42_H_72_O_14_	[M-H]-	Ginsenoside Rg1	Ginsenoside Rg1
mws1592	955.49	793.5	956.498	C_48_H_76_O_19_	[M-H]-	Ginsenoside Ro	Ginsenoside Ro
mws1666	945.54	783.5	946.55	C_48_H_82_O_18_	[M-H]-	Ginsenoside Re	Ginsenoside Re
mws4020	1077.59	945.6	1078.592	C_53_H_90_O_22_	[M-H]-	Ginsenoside Rb2	Ginsenoside Rb2
mws4022	783.49	459.5	784.497	C_42_H_72_O_13_	[M-H]-	20(S)-Ginsenoside Rg3	20(S)-Ginsenoside Rg3
mws4023	783.49	475.4	784.497	C_42_H_72_O_13_	[M-H]-	Ginsenoside Rg2	Ginsenoside Rg2
mws4026	637.43	475.1	638.439	C_36_H_62_O_9_	[M-H]-	Ginsenoside-F1	Ginsenoside F1
mws4027	655.44	457.2	654.434	C_36_H_62_O_10_	[M+H]+	Pseudoginsenoside RT5	Pseudo-Ginsenoside RT5
mws4028	621.44	459.3	622.444	C_36_H_62_O_8_	[M-H]-	Ginsenoside K	Ginsenoside K
mws4031	765.48	603.2	766.487	C_42_H_70_O_12_	[M-H]-	Ginsenoside Rk1	Ginsenoside Rk1
mws4035	783.49	621.5	784.497	C_42_H_72_O_13_	[M-H]-	Ginsenoside F2	Ginsenoside F2
mws4036	637.43	475.4	638.439	C_36_H_62_O_9_	[M-H]-	20(S)-Ginsenoside Rh1	20(S)-Ginsenoside Rh1
mws4038	769.47	475.3	770.482	C_41_H_70_O_13_	[M-H]-	Notoginsenoside R2	Ginsenoside R2
N1422	781.48	619.42	782.482	C_42_H_70_O_13_	[M-H]-	sanchirhinoside B	Ginsenoside B
N1633	961.54	799.49	962.545	C_48_H_82_O_19_	[M-H]-	Ginsenoside II	Ginsenoside II
N1669	1239.64	1239.64	1240.645	C_59_H_100_O_27_	[M-H]-	Notoginsenoside Rd	Ginsenoside Rd
N1757	1209.63	1209.63	1210.635	C_58_H_98_O_26_	[M-H]-	Ginsenoside Ra1	Ginsenoside Ra1
N1784	1107.6	945.53	1108.603	C_54_H_92_O_23_	[M-H]-	Ginsenoside	Ginsenoside B1
N1798	1193.6	1149.61	1194.603	C_57_H_94_O_26_	[M-H]-	Ginsenoside Mrb1	Ginsenoside Mrb1
N1838	769.48	637.43	770.482	C_41_H_70_O_13_	[M-H]-	Ginsenoside F3	Ginsenoside F3
N1859	811.49	769.47	812.492	C_43_H_72_O_14_	[M-H]-	sanchirhinoside A2	Ginsenoside A2

### 3.2 Principal component analysis of LSPN

**Figure 3:**
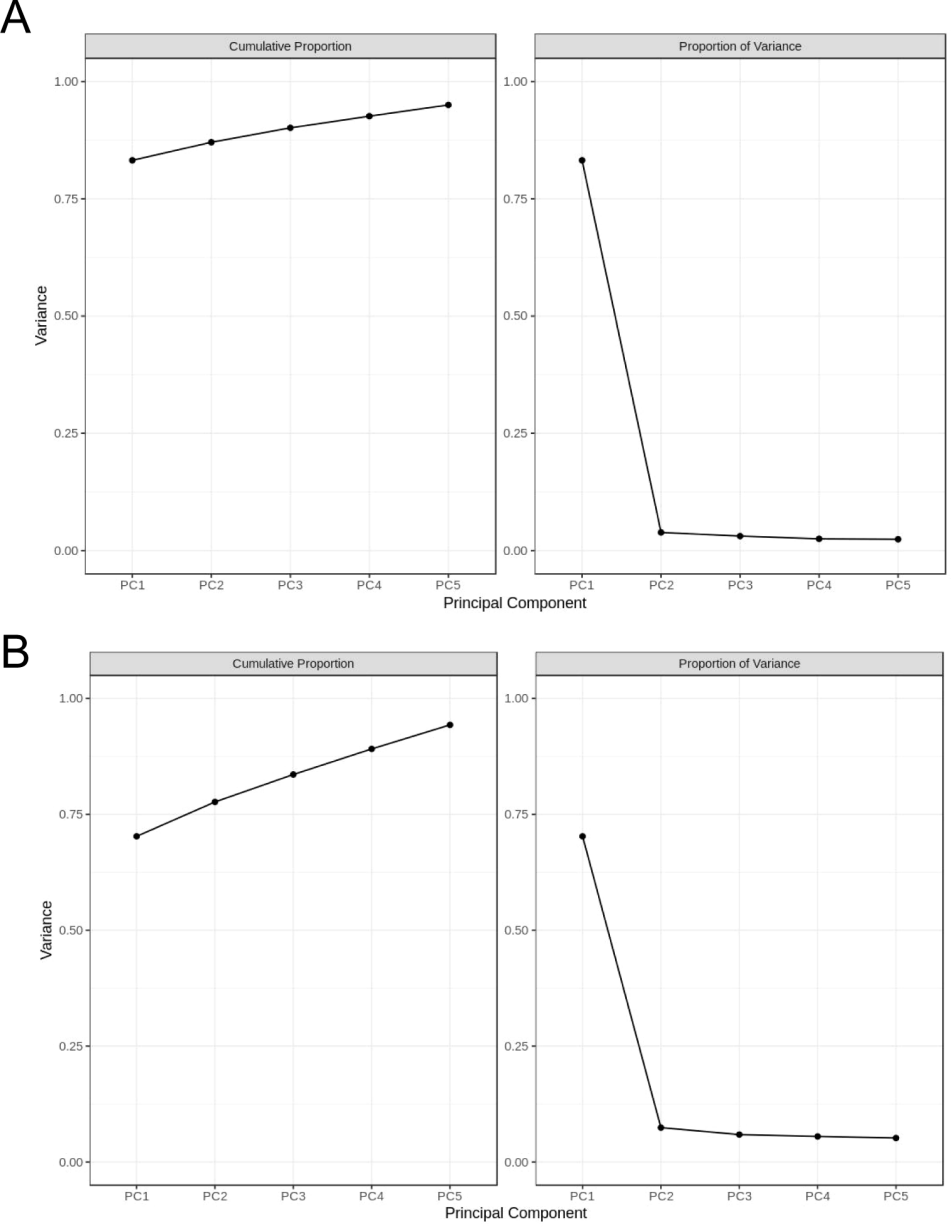
Principal component analysis of compounds and triterpenoid saponins. **(A)** Interpretable variation of the top 5 principal components of chemical compounds in each group; **(B)** Interpretable variation of the top 5 principal components of triterpene saponins in each group. The difference analysis of the first five main components shows the clarity and reliability of the two-dimensional analysis results.

**Figure 4:**
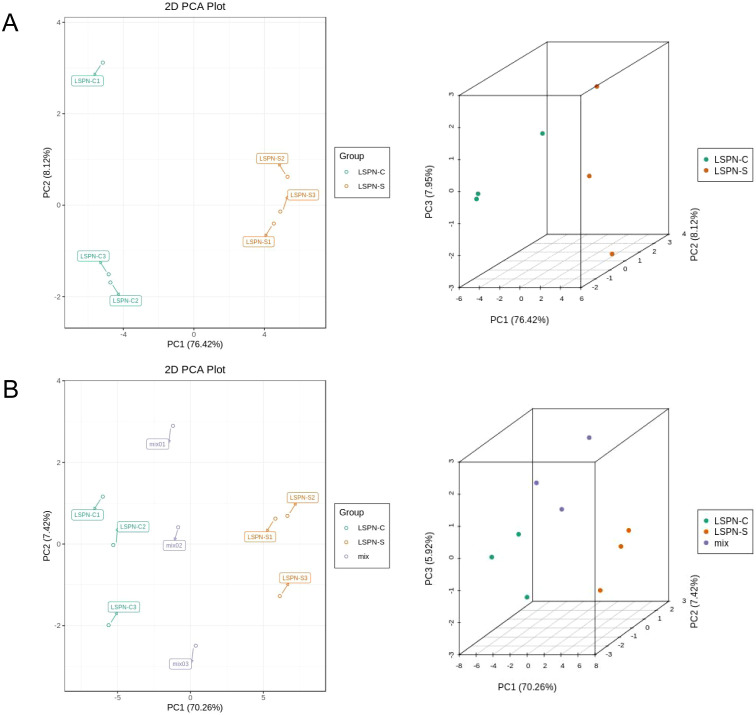
Two-dimensional and three-dimensional plots of principal component analysis of compounds and triterpenoid saponins. **(A)** PCA of the chemical compounds in each group; **(B)** PCA of the triterpene saponins contents in each group. The intra-group clustering phenomenon indicates that the same samples have good parallelism, and the distance between different groups in principal component analysis indicates the degree of similarity.

Through principal component analysis of all compounds (**Figure 3A**) and triterpene saponins (**Figure 3B**) in the samples (including quality control samples), it was found that principal component 1 (PC1) had the highest percentage of principal component variance, reaching 83% in **Figure 3**, and the other principal components had small percentages of variance (less than 4%); PC1 reached 70% in **Figure 4**, and the other principal components had small percentages of variance (less than 8%), which indicates that the two-dimensional principal component analysis can fully show the separation trend between and within each sample group, revealing whether there are differences in compounds and triterpenoid saponins between groups (Chen et al., 2013; Gu et al., 2024).

**Figures 4A, B** show the two-dimensional and three-dimensional plots of the principal component analysis of the compounds and triterpenoid saponins in the samples (including the quality control samples), respectively. As can be seen in **Figure 4**, the principal components analysis (PCA) plots of the compounds of Panax pseudoginseng stem and leaf LSPN extract (LSPN-S), Panax pseudoginseng stem and leaf control LSPN extract (LSPN-C) and the quality control samples showed clear distinctions between the groups and clustering within the groups, which indicated that there were some differences in compounds in the groups. In the PCA analysis of the triterpene saponins of the three samples, there was obvious intra-group aggregation of the groups on the first principal component (PC1), and the distinction was not obvious on PC2, which indicated that there were also some differences in the content of triterpene saponins among the groups.

### 3.3 Correlation analysis and cluster analysis

**Figure 5:**
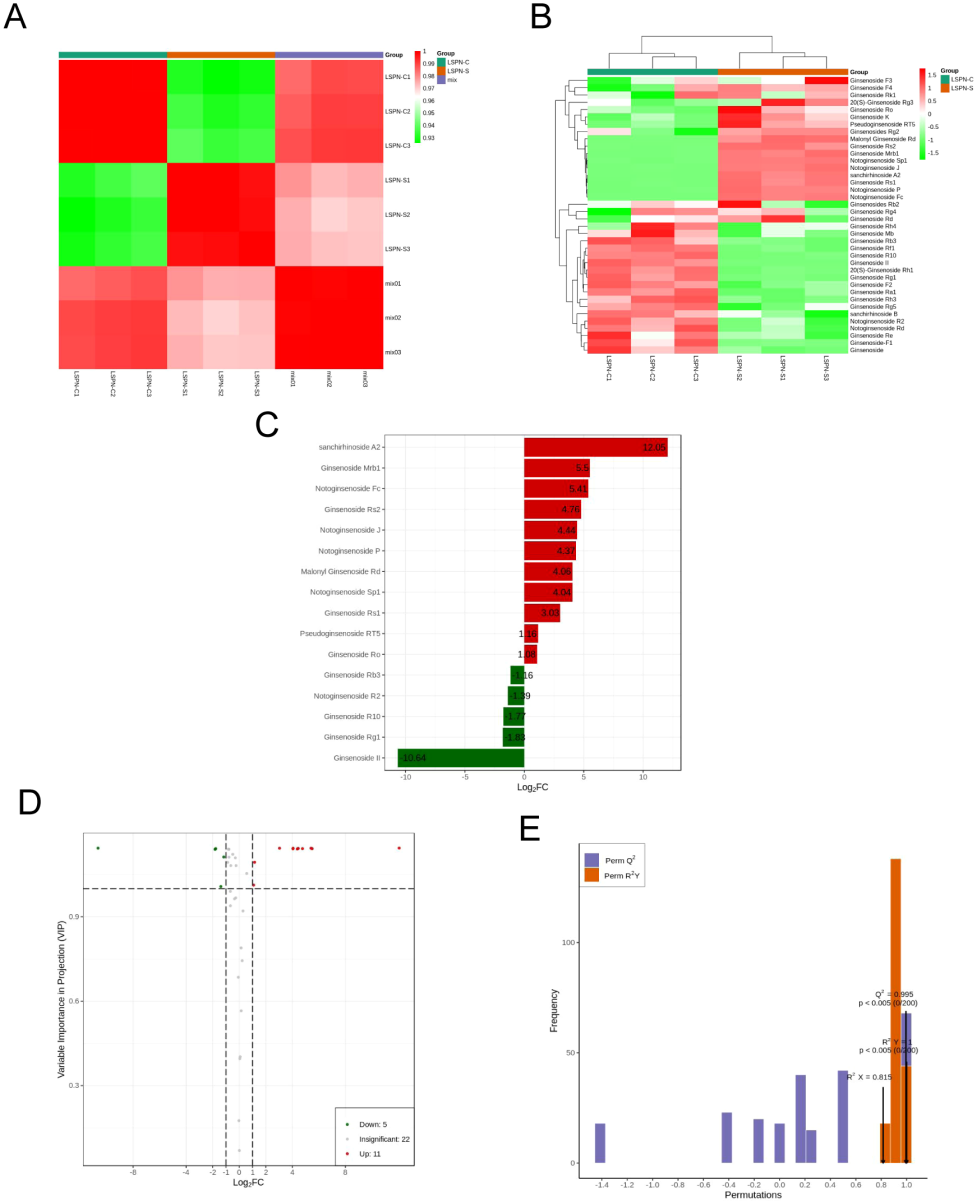
Triterpenoid saponins analysis. **(A)** Pearson correlation coefficient heat map of triterpene saponin content in each group; **(B)** Cluster diagram of triterpene saponin content in each group; **(C)** Histogram of fold change of different chemical compounds of triterpene saponins; Note: The abscissa is the base 2 logarithm of fold change, the ordinate is the differential chemical compounds, the red is the up-regulated differential metabolite, and the green is the down regulated differential chemical compounds. **(D)** Volcano map of differential chemical compounds of triterpene saponins; **(E)** The permutation of orthogonal partial least squares-discriminant analysis; Note: The abscissa is the model accuracy, and the ordinate is the frequency of the model classification effect. A color gradient from red to green indicates the degree of intergroup correlation, with higher values representing stronger correlations. B The color gradient from red to green reflects the clustering intensity of saponin content across different groups, with higher values indicating greater clustering. C Numbers represent the fold changes for upregulated and downregulated compounds. D Red represents 11 upregulated compounds, green indicates 5 downregulated compounds, and gray denotes 22 compounds showing no significant change. E P<0.005: Among 200 random permutation experiments, fewer than 1 (i.e., 0) models outperformed the OPLS-DA model.

The thermogram of pearson correlation coefficients for triterpene saponin contents in each group, shown in **Figure 5A**, had higher intra-group coefficients than inter-group, proving experiment reliability. The correlation coefficient between groups was over 0.92, indicating a significant positive correlation between the content of various triterpene saponins in LSPN-S and the triterpene saponin content in LSPN-C. Cluster analysis of triterpene saponin content, shown in **Figure 5B**, was normalized using the UNIT VARIANCE SCALING method. LSPN-C had higher content in the lower left corner, while LSPN-S had higher content in the upper right, suggesting some differences in triterpene saponin content between groups. However, correlation analysis in **Figure 5A** showed the overall difference was not large. Differences in triterpene saponin content of individual species need further analysis using the orthogonal partial least squares method for discriminant analysis.

### 3.4 Orthogonal partial least squares discriminant analysis

The above PCA analysis is not sensitive to variables with small correlations, while Partial Least Squares-Discriminant Analysis (PLS-DA) can solve such problems. Orthogonal Partial Least Squares-Discriminant Analysis (OPLS-DA) combines PLS-DA with the Orthogonal Signal Correction (OSC) method, where the information of the independent variables is decomposed into two categories: those that are correlated with the dependent variable and those that are not. After removing the uncorrelated differences, the discrepant variables can be filtered out. The results of the OPLS-DA calculations are detailed in **Table 3**. By comparing **Tables 2** and **3**, a total of 38 triterpenoid saponins were found to be contained in both groups, and all the species appeared in LSPN-S and LSPN-C, suggesting that LSPN-S and LSPN-C have the same composition. According to the data in **Table 3**, 11 of the 38 triterpenoid saponins were up-regulated in Panax ginseng stem and leaf LSPN extracts (LSPN-S) relative to the control LSPN extract (LSPN-C), 5 were down-regulated, and 22 others showed insignificant changes (fold change < 2). The bar charts of fold change for the different compounds are detailed in **Figure 5C**, and the volcano plots of all triterpene saponins in LSPN-S and LSPN-C are detailed in **Figure 5D**, which shows that the up-regulated compounds, in order of fold change, were: sanchirhinoside A2, Ginsenoside Mrb1, Notoginsenoside Fc, Ginsenoside Rs2, Notoginsenoside J, Notoginsenoside P, Malonyl Ginsenoside Rd, Notoginsenoside Sp1, Ginsenoside Rs1, Pseudoginsenoside RT5, Ginsenoside Ro; the down-regulated compounds, in order of fold change, were Ginsenoside Rb3, Notoginsenoside R2, Ginsenoside R10, Ginsenoside Rg1, and Ginsenoside II. From the validation plot of OPLS-DA (**Figure 4E**), Q^2^ is 0.995 with P less than 0.005, indicating that less than 1 (i.e., 0) of the 200 random permutation experimental models outperforms the present OPLS-DA model, and R^2^Y is 1 with P less than 0.005, indicating that less than 1 (i.e., 0) of the 200 random permutation experiments outperforms the present OPLS-DA model. Generally the model is best when P is less than 0.05, while the P of the present model is less than 0.005, which responds to the accuracy of the present model, and the above differences of triterpenoid saponins were discriminated accurately.

**Table 3:** VIP value and fold change of saponin compounds.

Index	Compounds	Substance	VIP	Fold Change	Type
P3625	Ginsenoside Rh3	Ginsenoside Rh3	1.11166	0.58520	insig
HJAP107	Ginsenoside Rh4	Ginsenoside Rh4	0.68500	0.95217	insig
mws4028	Ginsenoside K	Ginsenoside K	1.05384	1.47745	insig
Lmzp004417	Ginsenoside Rf1	Ginsenoside Rf1	1.14024	0.56527	insig
mws4036	20(S)-Ginsenoside Rh1	20(S)-Ginsenoside Rh1	1.13966	0.58402	insig
mws4026	Ginsenoside-F1	Ginsenoside F1	1.08178	0.64188	insig
P1561	Ginsenoside R10	Ginsenoside R10	1.14197	0.29255	down
mws4027	Pseudoginsenoside RT5	Pseudo-Ginsenoside RT5	1.09362	2.22982	up
P2705	Ginsenoside F4	Ginsenoside F4	0.78923	1.10442	insig
Lmzp005197	Ginsenoside Rg5	GinsenosideRg5	0.99023	0.63027	insig
mws4031	Ginsenoside Rk1	Ginsenoside Rk1	0.39585	1.02978	insig
P3979	Ginsenoside Rg4	Ginsenoside Rg4	0.06924	1.00516	insig
mws4038	Notoginsenoside R2	*Panax notoginseng* saponin R2	1.00750	0.38269	down
N1838	Ginsenoside F3	Ginsenoside F3	0.56574	1.10790	insig
N1422	sanchirhinoside B	*Panax notoginseng* saponin B	0.96381	0.78097	insig
mws4022	20(S)-Ginsenoside Rg3	20(S)-Ginsenoside Rg3	0.74374	1.16604	insig
mws4023	Ginsenosides Rg2	Ginsenoside Rg2	0.92055	1.21594	insig
mws4035	Ginsenoside F2	Ginsenoside F2	1.10923	0.81414	insig
mws1553	Ginsenoside Rg1	Ginsenoside Rg1	1.13979	0.28211	down
N1859	sanchirhinoside A2	*Panax notoginseng* saponin A2	1.14418	4234.51852	up
P3527	Notoginsenoside Sp1	*Panax notoginseng* saponin Sp1	1.14177	16.49864	up
N2170	Ginsenoside Rs2	Ginsenoside Rs3	1.14207	27.13280	up
P4021	Notoginsenoside J	*Panax notoginseng* saponin J	1.14282	21.77515	up
N2059	Ginsenoside Mb	Ginsenoside Mb	0.96710	0.83165	insig
mws1666	Ginsenoside Re	Ginsenoside Re	0.93908	0.63024	insig
Lmzp005427	Ginsenoside Rd	Ginsenoside Rd	0.40232	1.05067	insig
mws1592	Ginsenoside Ro	Ginsenoside Ro	1.01247	2.11972	up
N1633	Ginsenoside II	Ginsenoside II	1.14379	0.00063	down
N1971	Malonyl Ginsenoside Rd	Malonylginsenosides Rd	1.14325	16.64867	up
N1972	Notoginsenoside P	*Panax notoginseng* saponin P	1.14098	20.67109	up
mws4020	Ginsenosides Rb2	Ginsenoside Rb2	0.17552	0.98121	insig
MWKH438401	Ginsenoside Rb3	Ginsenoside Rb3	1.11221	0.44604	down
N1784	Ginsenoside	Ginsenoside B1	1.09336	0.54673	insig
N1874	Ginsenoside Rs1	Ginsenoside Rs1	1.14365	8.19290	up
N1798	Ginsenoside Mrb1	Ginsenoside Mrb1	1.14190	45.11052	up
N1757	Ginsenoside Ra1	Ginsenoside Ra1	1.12195	0.71227	insig
Lmzn004497	Notoginsenoside Fc	*Panax notoginseng* saponin Fc	1.14358	42.43626	up
N1669	Notoginsenoside Rd	*Panax notoginseng* saponin Rd	1.08166	0.84656	insig

All are the calculation results of the sample group with reference to the control group. “up” is marked when VIP ≥ 1 and Fold Change ≥ 2; “down” is marked when VIP ≥ 1 and Fold Change ≤ 0.5; “insig” is marked when VIP < 1 or 0.5 < Fold Change < 2.

**Figure 6:**
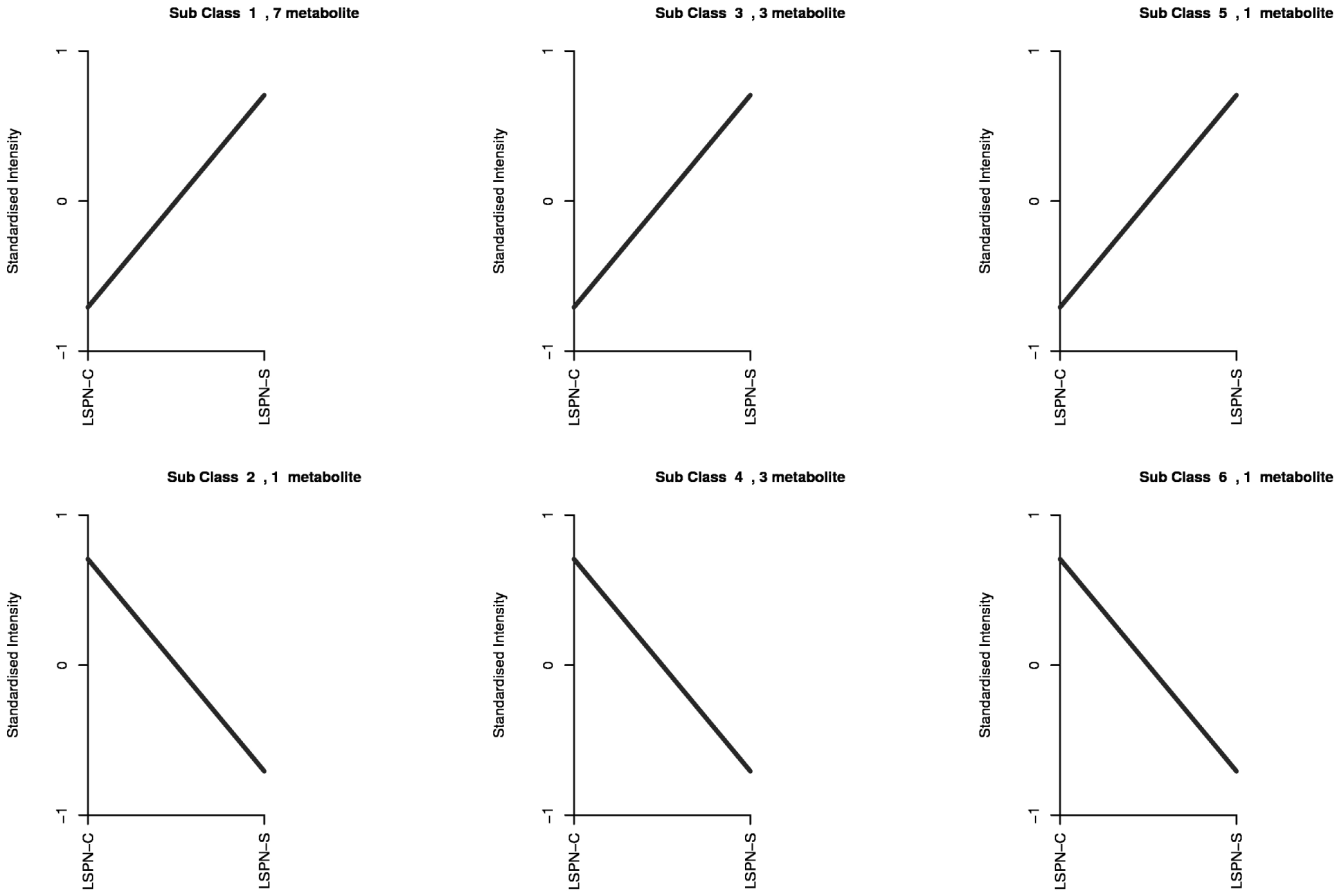
The K-means plot of differential chemical compounds. The abscissa is the sample name, and the ordinate is the normalized relative content of metabolites. The chemical compounds of Class 1 are pseudoinsenside rt5, notoginsenside SP1, ginsenoside RS2, malonyl ginsenoside Rd, notoginsenside P, ginsenoside RS1, ginsenoside mrb1; The chemical compounds of Class 2 is ginsenoside Rg1; The chemical compounds of class 3 are sanchirhinoside A2, Notoginsenoside J and Notoginsenoside Fc; The chemical compounds of Class 4 are ginsenoside R10, Notoginsenoside R2 and ginsenoside Rb3; The chemical compounds of Class 5 is ginsenoside ro; The chemical compounds of Class 6 is ginsenoside II.

The trends of the relative contents of each triterpenoid saponin differential compound in different samples after standardization and centering are shown in K-means analysis. Relative to LSPN-C, the relative contents of compounds of classes 1, 3, and 5 in the graph after standardization in LSPN-S are increasing; the relative contents of compounds of classes 2, 4, and 6 after standardization in LSPN-S are decreasing. This is consistent with the previous analysis, where K-means analysis was performed to categorize the 16 triterpenoid saponin-like differential compounds, and 6 classes were obtained (**Figure 6**).

## 4 Discussion

Currently, non-targeted plant metabolomics methods have been widely used in the study of traditional Chinese medicine, and such methods do not require chemical standards or prior knowledge of the chemical composition in the herbs to be tested. For example, Lele Li applied the RP-LC-MS method to analyze the saponin-like components in ginseng and western ginseng and distinguished ginseng and western ginseng on top of the three types of amino acids, saponins, and oligosaccharides, respectively, which expands the application of utilizing non-targeted plant metabolomics in the differentiation of plant components (Allwood et al., 2021; Li et al., 2022b; Li et al., 2023). The existence of matrix effect, detector saturation and other factors, non-targeted plant metabolomics in the application of a narrow linear range, quantification is difficult to accurately and other shortcomings, while a wide range of sexuality, reproducibility, sensitivity, etc., are the advantages of targeted plant metabolomics, but the need to use the standard, is not conducive to the acquisition of a more complete sample composition of the samples of the sample information is the disadvantage of targeted plant metabolomics methods (Han et al., 2023). Therefore, it is possible to choose which metabolomics method to be performed or to combine the two according to the research needs (Zhang et al., 2022).

The broadly targeted metabolomic technology used in this study was realized by Chen Wei et al (Chen et al., 2013). who built a library based on secondary spectral information (MS2 spectral tag (MS2T) library) from ESI MS/MS data. They established a stepwise multiple ion monitoring-enhanced product ion (stepwise MIM-EPI) strategy to build the MS2T library. The stepwise multi-ion detection method was used as a comprehensive scanning approach to trigger the acquisition of enhanced product ions. A broadly targeted metabolomics method was established by integrating the MS2T spectral library and other accessible multiple reaction monitoring information, which was able to quantitatively analyze 277 out of 698 metabolites in rice grains, and was suitable for metabolite difference analysis among different samples (Fraga et al., 2010). The overall saponin profile correlation (Pearson coefficient >0.92) between LSPN-S and LSPN-C suggests preserved pharmacological properties. Thermal processing-induced increases in rare ginsenosides (e.g., Rg3, Rs2) may actually enhance bioavailability, as these deglycosylated forms show better intestinal absorption (Kim et al., 2021). The balanced changes in protopanaxadiol (PPD)-type (e.g., Rb3) and protopanaxatriol (PPT)-type (e.g., Rg1) saponins maintain the optimal PPD: PPT ratio (1.2:1) associated with cardiovascular benefits while minimizing potential hypertensive effects. While this study focused on compositional analysis, prior toxicological data support the safety of “*P. notoginseng*” by-products. Acute toxicity tests in rats (LD50 >15 g/kg for stem/leaf extracts) and 90-day subchronic studies (NOAEL = 2 g/kg/day) revealed no organ toxicity (Su et al., 2024). The absence of hepatotoxic pyrrolizidine alkaloids—a concern for some herbal materials—was confirmed via UPLC-QTOF screening (Ma et al., 2022).

Beyond the statistical significance, the observed metabolomic differences between LSPN-S and LSPN-C are highly relevant from a pharmacological perspective. The strong overall correlation in saponin profiles (Pearson coefficient >0.92) indicates that the core pharmacological properties are largely preserved between batches. More importantly, the specific changes observed align with known structure-activity relationships. For instance, the thermal processing conditions for LSPN-S likely promoted the deglycosylation of major saponins, leading to the observed up-regulation of rare ginsenosides such as Rg3 and Rs2. This is not merely a chemical change; literature has consistently demonstrated that these rare ginsenosides exhibit enhanced bioavailability and more potent bioactivities, including superior anti-inflammatory, neuroprotective, and anti-cancer effects, compared to their glycosylated precursors (e.g., Rb1, Rg1). Therefore, the process-induced metabolic signature of LSPN-S may not represent a loss of quality but a shift towards a profile with potentially enhanced efficacy.

From the analysis of the results, it can also be found that this method was used in this study to study the stem and leaf saponins of Panax pseudoginseng, and the results also possessed excellent accuracy to detect the differences of triterpene saponin analogs between the samples and the control. The observed metabolomic differences between LSPN-C and LSPN-S (e.g., higher levels of ginsenoside Rg3 in LSPN-S) correlate with their divergent extraction conditions (higher temperature/longer duration), consistent with prior reports on thermal degradation of saponins (Bai et al., 2023). This underscores the utility of our batch selection criteria for detecting process-induced variability—a critical aspect of quality control in GMP-compliant production (Novoa et al., 2024).

The triterpene saponin-like constituents of Panax ginseng stem and leaves had a pearson correlation coefficient higher than 0.92 as a strong positive correlation compared with the control herb (**Figure 4A**), indicating that the contents of each triterpene saponin constituent in LSPN-S and LSPN-C were highly similar. Combining **Tables 2** and **3** reveals that there are 38 triterpenoid saponin compounds in both groups of extracts, of which 11 are up-regulated and 5 are down-regulated. The up-regulated compounds (e.g., sanchirhinoside A2, Ginsenoside Mrb1, Notoginsenoside Fc) have been previously associated with enhanced cardiovascular protection and anti-inflammatory effects (Zhao et al., 2025). Notably, the 45-fold increase in Ginsenoside Mrb1 may potentiate neuroprotective effects, as this compound has shown significant anti-apoptotic activity in neuronal cells (Zhao et al., 2018). The 16–27 fold increases in Notoginsenoside P and Malonyl Ginsenoside Rd are particularly relevant as these compounds contribute to the hemolytic index and bioavailability of the final product (Kim et al., 2018). Conversely, the down-regulated compounds included Ginsenoside Rb3 (0.45-fold), which is the pharmacopoeial marker for *Panax notoginseng* stems and leaves (DBS53/024-2017). While this reduction was statistically significant (VIP >1), the absolute content (2.1 mg/g) remained well above the minimum pharmacopoeial requirement (1.5 mg/g), suggesting maintained efficacy. The significant reduction in Ginsenoside Rg1 (fold change = 0.28, VIP = 1.14) warrants consideration, as this compound contributes to platelet aggregation inhibition (Yang et al., 2022), though its content (1.8 mg/g) remains within the therapeutic window established in prior clinical studies (Bi et al., 2021; Povydysh et al., 2021). These findings align with prior reports that thermal processing can convert major saponins into more bioactive rare ginsenosides without compromising safety. The increased levels of malonylated saponins (e.g., Malonyl Ginsenoside Rd) may enhance water solubility and absorption, as demonstrated in pharmacokinetic studies (Lehto et al., 2019; Liu et al., 2022).

While 12 saponins were unequivocally confirmed (Level 1), the remaining 26 identifications (Level 2) rely on high-resolution spectral matching. Although our stringent thresholds (cosine ≥ 0.8, mass error ≤10 ppm) minimize false positives, isomeric saponins (e.g., Rg2/Rg6) could not be resolved without standards. Future work should prioritize isolation of these compounds for absolute confirmation. In addition, the observed RSD values (<15% intra-day, <20% inter-day) for major saponins demonstrate excellent method precision, supporting the reliability of our batch-to-batch comparisons. The low LOD/LOQ values (0.01-0.15 μg/mL) confirm adequate sensitivity for detecting process-induced variations in saponin profiles. It is important to acknowledge a limitation of this study: while we evaluated batch-to-batch variability, our design was cross-sectional and did not assess the potential impact of seasonal variability in the raw materials or the long-term temporal stability of the finished LSPN product. The saponin profile of *Panax notoginseng* stems and leaves can be influenced by factors such as harvest time and growing conditions. Future studies specifically designed to incorporate raw materials from different seasons and to include accelerated stability testing of the final product will be essential to develop a more comprehensive quality control strategy that ensures consistency throughout the product’s shelf life.

Control herbs are national drug standard substances used for the identification of Chinese herbal medicines and proprietary Chinese medicines after appropriate treatment, which can be used for the identification analysis of thin-layer chromatography and characterization, etc. They are mainly used for qualitative identification, and the quantitative analysis of the components of Chinese herbal medicines mainly relies on the chemical control of Chinese herbal medicines. Therefore, whether the content of raw materials and control herbs is identical or not is not the main basis for the evaluation of the quality of herbs. The present study showed that LSPN-S and LSPN-C have the same composition and highly similar contents, in which the content analysis of ginsenosides Rb3 and Rb1, the main components in LSPN-S, will be further investigated.

## 5 Conclusions

Through the application of widely-used targeted metabolomics techniques, we conducted in-depth analysis and exploration, further identifying the *Panax notoginseng* stem and leaf medicinal materials we selected. Additionally, we thoroughly analyzed the main components of LSPN. This process not only deepened our understanding of the *Panax notoginseng* stem and leaf medicinal materials and LSPN but also revealed some previously unknown information. We hope that these new findings and insights will provide novel ideas and perspectives for subsequent clinical research, promoting in-depth progression of related studies.

For clinical translation, future work should address: (1) Standardization of extraction parameters to minimize batch variability (RSD <15% for key saponins); (2) Phase I trials to validate human safety, particularly for malonyl-saponins which lack clinical data; and (3) Ecological assessments to ensure large-scale stem/leaf harvests do not disrupt soil nutrient cycles. These steps align with WHO guidelines for herbal medicine development (WHO/EDM/TRM/2022.1).

In conclusion, we have established and validated a broadly targeted metabolomics approach for assessing the consistency of LSPN across production batches. While this study provides a crucial foundation for controlling manufacturing variability, future research must expand to include temporal and seasonal dimensions to fully guarantee product quality from raw material to expiry.

## Data Availability

The original contributions presented in the study are included in the article/**Supplementary Material**. Further inquiries can be directed to the corresponding author.
