# Supplementary material for: Analysis of the main components of LSPN based on broadly targeted metabolomics technology
Source: Front Plant Sci. 2025 Oct 22;16:1618168. doi: 10.3389/fpls.2025.1618168 (PMC12587676; doi:10.3389/fpls.2025.1618168)

Analysis of the main components of LSPN based on broadly targeted metabolomics technology

Feng Qin [a,c] , Yuyong Chen [b] , Dada Wang [b] , Jingui Li [c*] and Shanyuan Zhu [a*]

[a] Jiangsu Provincial Key Laboratory of Veterinary Bio-pharmaceutical High-tech Research, Jiangsu Agri-animal Husbandry Vocational College, Taizhou, Jiangsu, China

[b] College of Food Science and Technology, Jiangsu Agri-animal Husbandry Vocational College, Taizhou, Jiangsu, China

[c] College of Veterinary Medicine, Yangzhou University, Yangzhou , Jiangsu, China

Author for correspondence Shanyuan Zhu, PhD, Jiangsu Provincial Key Laboratory of Veterinary Bio-pharmaceutical High-tech Research, Jiangsu Agri-animal Husbandry Vocational College, 8 Fenghuang East Road, Taizhou 225300, China (e-mail: jstzmy922@163.com).

Supplementary Figures

Figure S1. Mixed-sample mass spectrometry analysis of total ion flow plots. **A** Positive Ion Mode; **B** Negative Ion Mode.Note: Each different color chromatogram peak represents a detected compound.

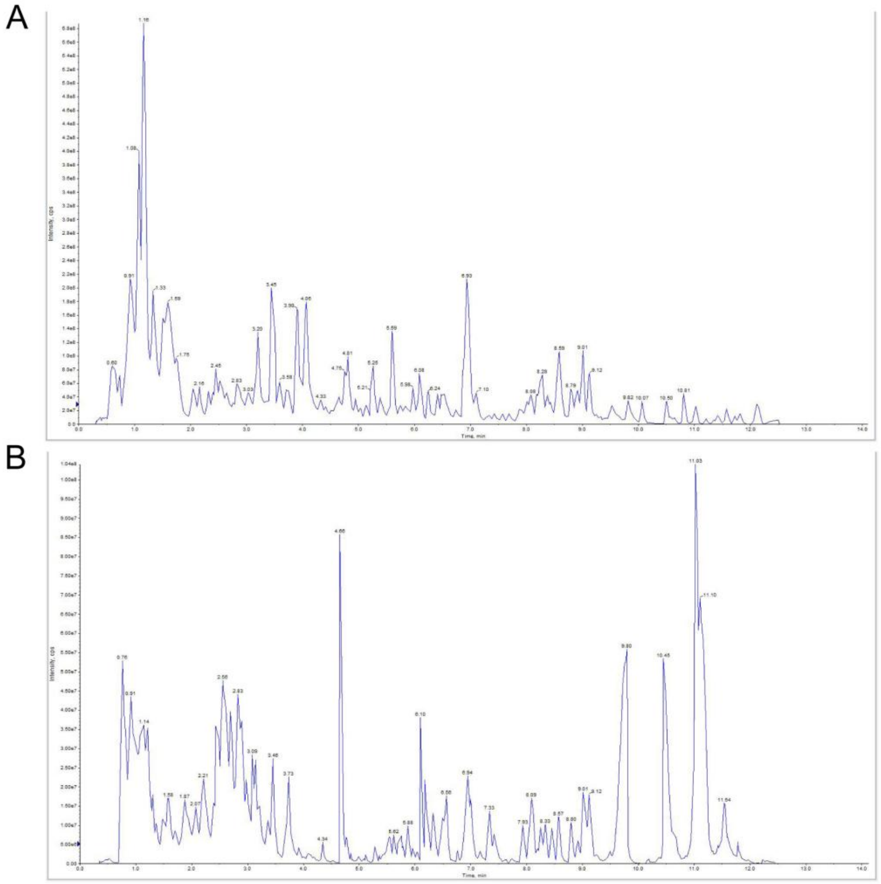

Figure S2. Multi peak diagram of MRM chemical compounds detection of LSPN mixed sample. **A** Positive Ion Mode; **B** Negative Ion Mode. Note: The abscissa is the retention time (min) of the chemical compound, and the ordinate is the ion current intensity (cps) of the chemical compound.

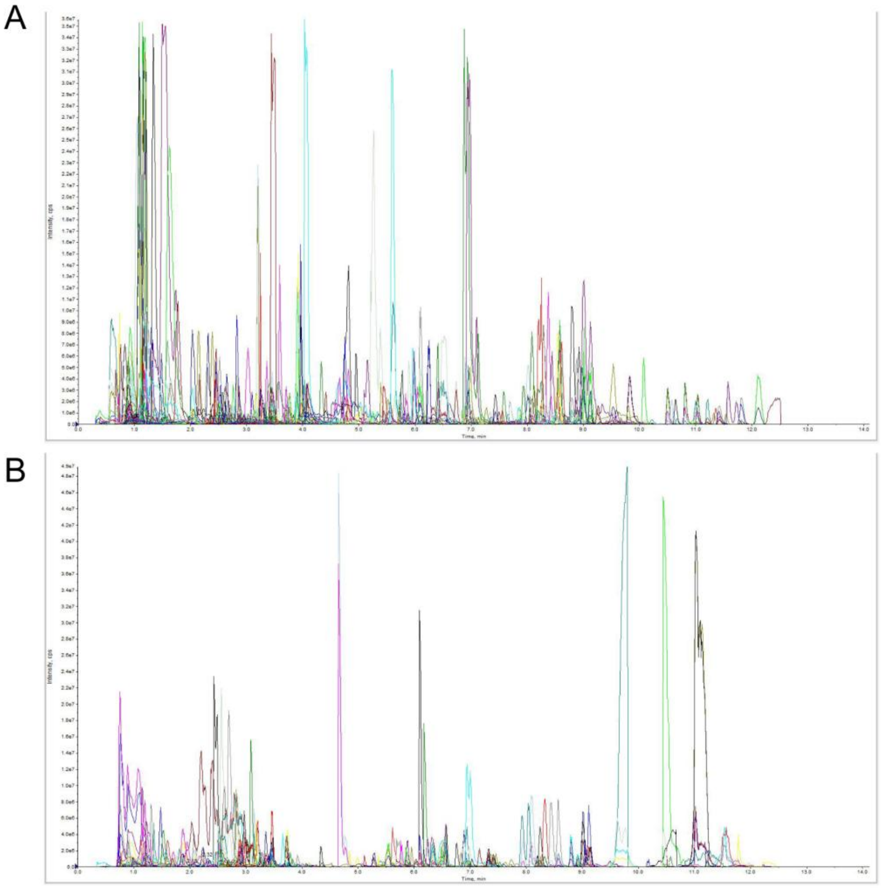

Figure S3. QC sample mass spectrometry detection TIC overlap chart. **A** Positive Ion Mode; **B** Negative Ion Mode. Note: The higher the overlap, the higher the stability of the mass spectrum signal

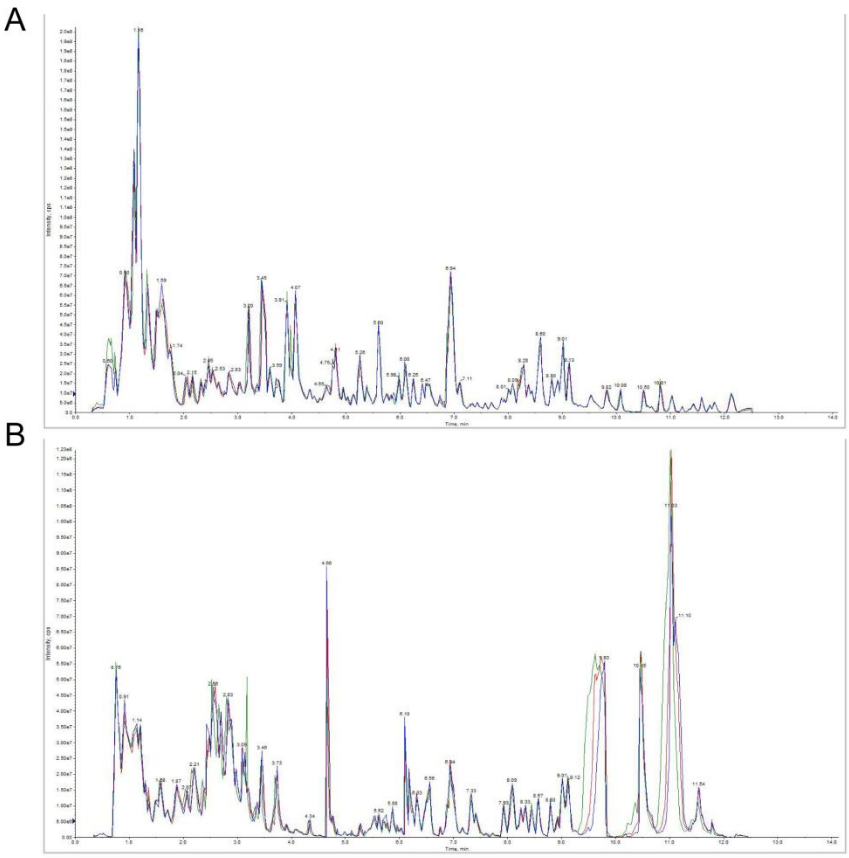

Supplement: Supplementary file 1 [file Image1.pdf]
